# Supplementary figures and images for: Mental health and gender-based violence: An exploration of depression, PTSD, and anxiety among adolescents in Kenyan informal settlements participating in an empowerment intervention
Source: PLoS One. 2023 Mar 29;18(3):e0281800. doi: 10.1371/journal.pone.0281800 (PMC10057741; doi:10.1371/journal.pone.0281800)

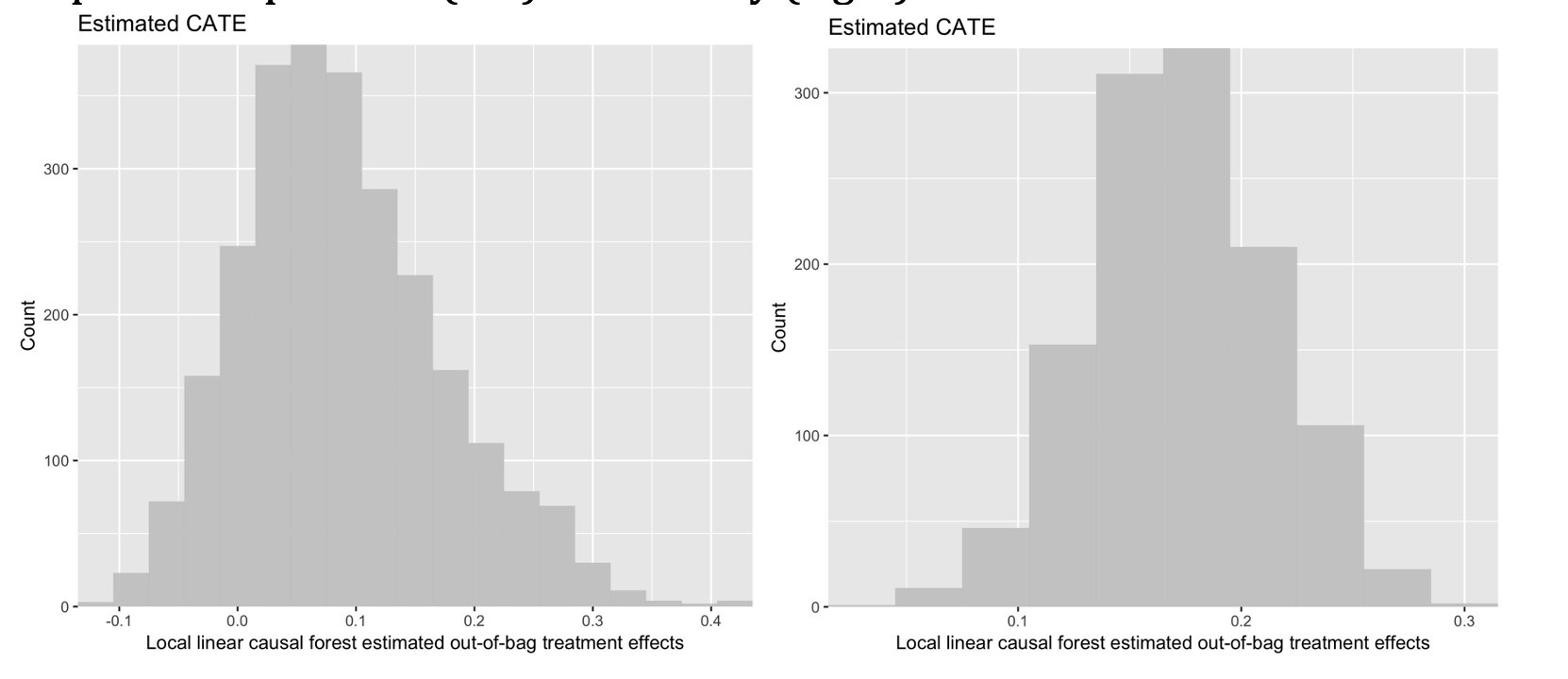

Supplement: S1 Fig — We consider depression (left) and anxiety (right). (TIFF) [file pone.0281800.s001.tiff]
