# Supplementary material for: Mental health and gender-based violence: An exploration of depression, PTSD, and anxiety among adolescents in Kenyan informal settlements participating in an empowerment intervention
Source: PLoS One. 2023 Mar 29;18(3):e0281800. doi: 10.1371/journal.pone.0281800 (PMC10057741; doi:10.1371/journal.pone.0281800)
Supplement: S1 Table — These estimates are not weighted by probability of dropout during the study. (DOCX) [file pone.0281800.s002.docx]

| **Group** | **PTSD** | **95% CI** | **Depression** | **95% CI** | **Anxiety** | **95% CI** |
| --- | --- | --- | --- | --- | --- | --- |
| *Overall* | 9.1% | (8.1%, 10.3%) | 6.7% | (5.0%, 6.9%) | 11.2% | (7.0%, 11.9%) |
| *Male* | 9.4% | (7.3%, 11.5%) | 4.5% | (3.2%, 6.2%) | 12.6% | (9.4%, 16.9%) |
| *Female* | 9.0% | (8.1%, 10.1%) | 8.1% | (7.0%, 9.0%) | 14.8% | (13.0%, 16.7%) |
| *Female,* $R_{0}$ *=0* | 8.3% | (7.1%, 9.2%) | 7.3% | (6.3%, 8.1%) | 13.8% | (11.6%, 15.4%) |
| *Male,* $R_{0}$ *=0* | 9.6% | (7.8%, 11.6%) | 4.2% | (2.9%, 5.6%) | 11.8% | (8.0%, 15.3%) |
