# Supplementary material for: Mental health and gender-based violence: An exploration of depression, PTSD, and anxiety among adolescents in Kenyan informal settlements participating in an empowerment intervention
Source: PLoS One. 2023 Mar 29;18(3):e0281800. doi: 10.1371/journal.pone.0281800 (PMC10057741; doi:10.1371/journal.pone.0281800)
Supplement: S2 Table — Prevalence and 95% bootstrap confidence intervals are displayed for each mental health outcome and each subgroup, without IPW. (DOCX) [file pone.0281800.s003.docx]

| **Group** | **PTSD** | **95% CI PTSD** | **Depression** | **95% CI depression** | **Anxiety** | **95% CI anxiety** |
| --- | --- | --- | --- | --- | --- | --- |
| *Overall* | 9.1% | (8.1%, 10.3%) | 6.7% | (5.0%, 6.9%) | 11.2% | (7.0%, 11.9%) |
| *Female-T* | 8.6% | (7.1%, 9.6%) | 7.3% | (6.0%, 8.6%) | 14.5% | (11.7%, 17.2%) |
| *Female-C* | 9.5% | (7.7%, 10.9%) | 9.1% | (7.7%, 10.3%) | 15.2% | (12.5%, 18.0%) |
| *Female-T with* $R_{0}$ *=0,*$R_{1}$ *=1* | 30.4% | (23.5%, 39.6%) | 24.5% | (19.2%, 30.5%) | 35.0% | (20.0%, 50.0%) |
| *Female-C with* $R_{0}$ *=0,*$R_{1}$ *=1* | 25.5% | (16.3%, 32.4%) | 17.4% | (11.8%, 25.3%) | 35.0% | (20.5%, 51.4%) |
| *Female-T with* $R_{1}$ *= 0* | 7.2% | (5.9%, 8.5%) | 6.1% | (4.6%, 7.4%) | 13.5% | (11.1%, 15.9%) |
| *Female-C with* $R_{1}$ *= 0* | 8.3% | (6.9%, 9.9%) | 8.1% | (6.8%, 9.2%) | 13.9% | (11.2%, 16.4%) |
