# Supplementary material for: Mental health and gender-based violence: An exploration of depression, PTSD, and anxiety among adolescents in Kenyan informal settlements participating in an empowerment intervention
Source: PLoS One. 2023 Mar 29;18(3):e0281800. doi: 10.1371/journal.pone.0281800 (PMC10057741; doi:10.1371/journal.pone.0281800)
Supplement: Attachment — Submitted filename: Response to Reviewers.docx [file pone.0281800.s004.docx]

June 20, 2022

We thank the editor and the reviewer for their helpful comments! Responses to each individual point are included in the table below.

| Comments from editor: | Author response |
| --- | --- |
| 1. Please ensure that your manuscript meets PLOS ONE's style requirements, including those for file naming. | We have updated the formatting of the title page and manuscript. All file names should now have the correct naming convention and file type. |
| 2. We note that the grant information you provided in the ‘Funding Information’ and ‘Financial Disclosure’ sections do not match. When you resubmit, please ensure that you provide the correct grant numbers for the awards you received for your study in the ‘Funding Information’ section. | Thanks for pointing this out! The information should match now. |
| 3. If there are ethical or legal restrictions to sharing your data publicly, please explain these restrictions in detail. | We cannot submit the real data for privacy purposes, as the data describes mental health and sexual violence history, and hence is incredibly sensitive. We cannot risk any individual being personally identified or any privacy compromised by releasing this data not in aggregate. |
| 4. Please include your full ethics statement in the ‘Methods’ section of your manuscript file. In your statement, please include the full name of the IRB or ethics committee who approved or waived your study, as well as whether or not you obtained informed written or verbal consent. If consent was waived for your study, please include this information in your statement as well. | Added information on clinical trial registration, IRB approval, and consent to the Ethics section. |
| 5.  Please upload a copy of Figure 3, to which you refer in your text on page 15. If the figure is no longer to be included as part of the submission please remove all reference to it within the text. | Updated Figures and text so that the text only refers to relevant figures included. |
| 6. Please include captions for your Supporting Information files at the end of your manuscript, and update any in-text citations to match accordingly. Please see our Supporting Information guidelines for more information: [http://journals.plos.org/plosone/s/supporting-information](https://nam06.safelinks.protection.outlook.com/?url=http%3A%2F%2Fjournals.plos.org%2Fplosone%2Fs%2Fsupporting-information&data=05%7C01%7Crfriedberg%40linkedin.com%7Ca6ad641fe7ca4458850d08da4fe082ef%7C72f988bf86f141af91ab2d7cd011db47%7C1%7C0%7C637910123084414236%7CUnknown%7CTWFpbGZsb3d8eyJWIjoiMC4wLjAwMDAiLCJQIjoiV2luMzIiLCJBTiI6Ik1haWwiLCJXVCI6Mn0%3D%7C3000%7C%7C%7C&sdata=ouFMbEO%2BZjaeqeOEYgPi3zi0LRh3LspDBuZSRS2E0l8%3D&reserved=0). | We included a supporting information section with captions for the appropriate figure and tables. |
| Comments from reviewer #1: | Author response: |
| Abstract methods: need details for the study design and statistical methods. The statistical analysis is not clearly written. | We thank the reviewer for many helpful comments and suggestions! Responses to the individual points are included below. |
| Objective 1:  For table 2, clarify whether the outcomes are baseline or change. The mixed model was not mentioned in method section of Objective 1 (mentioned only in the title). | Added a sentence noting that the results are at follow-up, but aggregated across treatment and control arms, and noted this in the methods section for objective 2 (having moved table 2 to this section). |
| Why separate female and male? Gender can be added as a covariate and its interactions with other covariates can be evaluated. | We focus on female students here because we have much more data in this population because the original RCT was focused on sexual violence prevention methods for female students. We have relatively limited data on male students, and not enough to reliably model full interaction effects. This would be a worthwhile direction for future research. |
| Was log of score used? Normality tested? | There is a large right tail in the data, hence we used a log transformation to manage the outliers. |
| More important of all, better use logistic regression to model prevalence of PTSD, depression and anxiety as they are the primary outcomes for this objective. | Note that these scores are not a binary, and each student is scaled on a score. While we could model a logistic regression, we felt it was reasonable to use a linear model to understand the effect of prior rape, socioeconomic status, etc., on the sliding scale. |
| Further, Table 2 does not seem to belong here as the objective is for mental health prevalence, not associations with baseline characteristics. | We agree and moved Table 2 to the following section. |
| Objective 2:  What model did you use for GBV comparisons? Better use logistic regression rather than “Descriptive analysis”. GBV can be evaluated in the model. Need odds ratios and p values. | For GBV comparisons, we consider prevalence in groups with and without history of GBV, and compare confidence intervals; we also use a mixed model to estimate the effect of GBV on the continuous PTSD, anxiety, and depression scores. |
| Objective 3:  The “mixed” model mentioned in the method section does not match the results presented in Table 3. The method section mentioned “outcomes were log-transformed” but the outcomes in Table 3 are all dichotomous.  To evaluate the intervention effects, the analysis should focus on the comparisons between intervention and control. Logistic regression is more appropriate for dichotomous outcomes. GBV with other characteristics can be added as covariates. | Note that the outcomes are not dichotomous but measured on a scale; when evaluating prevalence, we use clinically determined cutoffs to make the outcome binary. But in other analyses, we prefer to leave the natural continuous variable as it is. |
| Objective 4:  How are low, mid and high-CATE determined? Are those cutoff points clinically meaningful? | The cutoff points were determined by quantiles, are are not clinically significant. |
| Predictors in Table 4 need to be clearly described. | Thanks for pointing this out! Added descriptions of each variable. |
| Need rationale to use log-transformation of outcomes. Does it improve normality? | Yes, it improves normality due to outliers and a long right tail. |
| Why bootstrap? As this is a large study. | The confidence intervals from lme4 are typically not as reliable as bootstrap confidence intervals. |
| Flow charts: clarify N is # of schools. | We removed the flow charts, as they primarily correspond to the original RCT and are available in that paper. Happy to put them back in if that’s preferred. |
