# Supplementary material for: Mental health and gender-based violence: An exploration of depression, PTSD, and anxiety among adolescents in Kenyan informal settlements participating in an empowerment intervention
Source: PLoS One. 2023 Mar 29;18(3):e0281800. doi: 10.1371/journal.pone.0281800 (PMC10057741; doi:10.1371/journal.pone.0281800)
Supplement: Attachment — Submitted filename: Response to reviewers.docx [file pone.0281800.s006.docx]

We want to thank the editor and the reviewers for their helpful comments. We have revised the manuscript to add more details on the study design and methods, clarify terminology, and expand on the implications of the study findings. We include a detailed response to the comments below.

Reviewer 2.

| Minor comment, at the end of the background section, line 93, “these communities”, which communities are you referring to? | These communities refers to the informal settlements of Nairobi; we have rephrased to clarify. |
| --- | --- |
| Currently, the study aims are described in the method section. I suggest moving them to the end of the background section in order to improve the flow of the text. | This is a great suggestion (and several reviewers noted that the study aims needed to be stated earlier). We moved the introduction of the study aims to the background section, and kept the more detailed material in the methods section. |
| In the method section, there is now description of the flow of participants/schools, e.g. number of dropouts. I understand that there was previously a flowchart describing this? I suggest to put it back. | Good point - we have put this chart back, thanks for this catch. |
| In the method section, please describe how many classes were included in the study. | We included one class per school; noted in the methods section. |
| Were the schools randomized based on a 1:1 allocation ratio? Was the baseline assessment conducted prior to or after randomization? Please specify. | Baseline data was collected and analyzed after a matched-pairs randomization; we added this note to the methods section (also see reference 29). |
| In the method section, where you describe the mental health measures, please describe how GBV was assessed? Was this assessed with a scale or single items? Can you provide an example of such items? It is now briefly described in the analysis objective 2 section but I suggest to also add it here in order to improve the flow of the text. | This makes sense given the flow of the paper, thanks for pointing it out. We moved the description of GBV history assessments earlier and added an example of a survey question. |
| In the method section, more information could be provided on where the data collection took place. At the same location as where the CRT took place? I assume that the adolescents completed the measures on their mobile phones/ computers? How long approximately did the data collection take? | We added a note that data were collected within the school participating in the CRT. The data were collected contemporaneously with the data for the CRT onsite, logged, and then entered online. Students in these schools could not be assumed to have access to mobile devices or computers, nor did local stakeholders consider it safe for team members to bring them onsite (e.g. experience with previous violent muggings). |
| In Tables 1 and 3, merely % of participants is provided. Is it possible to add N and total N to these tables? It would make them more informative. | Note that our estimates are weighted to account for population imbalances, so we anticipated that the N and total N might be more confusing than helpful; we do include total N in the CONSORT flow diagram. We include confidence intervals to help readers understand how tight these estimates are. |

Reviewer 3.

| A strength of the manuscript is the statistical analyses. There are however some assumptions or decisions made about the data that requires more clarification in the manuscript.  The manuscript will benefit from the inclusion of a diagram to help depict the timelines of the research and the assessment points. As the research has multiple components the manuscript lacks some flow in my opinion. I have tried to comment on this in the attached track changes documents. | Thanks for noting this- totally agree! We have added the diagram. |
| --- | --- |
| Changes tracked in attachment. | We have implemented the changes you suggest in the attached manuscript. Thank you very much for the careful reading of our manuscript! We will also respond to comments in the document here. |
| Why when one is evaluating an intervention will one only include mental health measures only to the final data collection point? | These measures were included as a follow-up to an RCT aiming to understand GBV prevention, which is why we only have them at follow-up. We would recommend for future research to include them at baseline. This is also why we only have the measures at the 2-year follow-up point (to address a later, related comment). |
| I think the authors have to think carefully how the use the term GBV and sexual assault and rape. In the manuscript it seems as if the term rape and GBV are used interchangeably. Rape or sexual assault is a only a part of GBV. When looking at the sample of males and females the key is that the rape/sexual assault was perpetrated across gender to be deemed GBV. So did the authors control for the gender of the perpetuator? We know that the majority of perpetuation against males and females are from male offenders. Did the study also look at other areas of GBV such as physical, economical and psychological harm? | This is an incredibly important and thoughtful point - thanks so much for bringing this up. We adjusted the language throughout our study to be specific about when we discuss rape, versus when we discuss GBV on broader terms. |
| I think the authors can improve on the methods section. They can think about headings such as procedure, assessment instruments, i also think a consort diagram or some type of diagram that describe the time lines of assessments and the the separation of the parts of the study, assessing prevalence rates and the effect of an intervention. A It will just improve the flow and the ease of reading for readers. | Thanks for including this note. We have received similar feedback across the reviews and worked to add CONSORT flow and make the section more accessible for readers! |
| why only female students and not also male students exposed to sexual assault? | The intervention focused on female students due to high prevalence of sexual assault and rape against adolescent female individuals in this population; exploratory programs to support male students were included in the RCT and should be explored more in the future. |
| explain how you decided on this solution and the possible implications. When these students dropped out, did they receive an exit interview and what was assessed at these points. The number of drop outs can also be shown on a timeline/consort diagram? To help readers understand the flow of participants in the study. | Unfortunately, students who dropped out would not be able to have exit interviews - this is often because they have moved schools or neighborhoods. This is common in informal settlements and very difficult to track for study purposes. We do however include the CONSORT diagram to help show the patterns of drop-out over time. |
| Analysis objective 3: explain why these covariates were chosen | These were previously identified as important features, and we added the reference here. |
| You will have to refer to the original study in this manuscript in more detail. You will have to explain the conditions associated with random assignment. | This is a great point; another reviewer also asked for more detail. We have added more context around the original randomization. |
| this between group comparison without an in group comparison is not ideal and it is most unfortunate that these measures were not done at baseline. This does not allow for a between group comparison at baseline and does raise a concern around comparing these measure between groups at FU | We completely agree and would absolutely suggest that measures be collected at baseline and follow-up. |

Reviewer 4.

| Abstract: Please state confidence intervals correctly (see CONSORT) | Rewrote confidence intervals according to CONSORT guidelines. |
| --- | --- |
| Please state statistical values in “findings”, at least p-values | We do not include p-values from the mixed effects model because there is not full clarity on calculating these and they can be misleading. The confidence intervals, however, can be reliably computed. |
| First sentence in conclusion can already be read in findings | Good point! We have removed this sentence. |
| Background: Please define and explain ESDs | We added a description for ESD programs after the first introduction of the term. |
| The background section is very short and really different paragraphs that don’t “make a story” | We have added some transitions and tried to expand this section so that the links and story are more clear. |
| The significant gap in the literature is not well described | This is true; it’s a bit difficult to point to an absence in the literature for such an understudied population (eg. these are the first results we know of that estimate prevalence of the mental health conditions we discuss in this population.). We have added some language noting the uniqueness of the current research to call attention to the dearth. We are quite open to further elaboration on this point. |
| Methods: Please cite the RCT when first mentioning it (registration number, main paper, ….) | We added a citation to the intervention when first mentioned, and moved the ethics section (which introduces registration number, etc.) earlier in the paper to come before the methods section. |
| Introduce abbreviations only once | Good catch; we removed extra definitions for abbreviations. |
| Aims of the current study need to be described in the background/ introduction section. Please describe more literature to each goal and state hypothesis | Thanks for the note; we moved some discussion of the study aims to be much earlier in the background section. Please note that we did not have specific hypotheses for the goals here: the goal was to asses prevalence and responses to the intervention without presuppositions about the outcomes, so we do not want to add hypotheses that we did not originally state. |
| Please insert the common headings (sample, intervention,…) | We added the headings; thanks for the suggestions. The methods section hopefully is much more organized. |
| Please describe measures more detailed with cut-off values | We include the cut-off values for each measure at line 154; we chose these by applying standards from the literature on moderate-to-severe conditions and did not expand on those existing results. |
| Please add the RCT flow-chart to better understand the high and systematic drop-out (I think this had been taken out afterwards? There is a crossed-out sentence on page 10) | We added this chart back, thanks for the note! |
| Please describe handling of missings | We address missing data in several ways. First, for students who dropped out, we use inverse probability weights to upweight the students who remained in the study who were most similar to them. Among the students who stayed for the duration of the study, we dropped missing observations. We have added a sentence addressing this before Objective 1. |
| I don’t understand the new sentence on page 8 | We rephrased this sentence for clarity. |
| Why was school not included as a control variable? The analysis could possibly also be implemented in a hierarchical structure | We adjust for school, accounting for its variation, by including schools as a random effect (Objective 3 section), because we can control for differences across schools without modeling them directly. This approach tends to produce valid standard errors, while maximizing precision in estimation of the “effects of interest.” Said another way: In this study, we take the stance that the individual-level effects are of interest, and the school-level effects are “nuisance parameters” (in the statistical sense). Statistically, we have models that can be described as using “hierarchical structure” but we’ve focused on student-level variation. |
| Discussion: Please discuss the finding that female participants “only” report higher depression but no PTSD and anxiety, as this is not in line with the literature (p.18 top) | We noted that future work might consider this pattern in a larger study, as it is difficult to attribute the negative finding to different patterns here vs. sample size and power (also discussed in the limitations section). |
| In the discussion section it is again not a coherent narrative but only sentences and paragraphs on a finding each | We have tried to smooth out this section and clarify transitions/movement between paragraphs. |
| The conclusion on potential combined interventions (p.18-19) is not based on the results, please rephrase | The suggestion included here is based on the results linking gender-based violence history (in addition to our results, references 46, 48, 49), and on the citation (50). |
| The discussion could benefit from more clinical and political implications of the result | We are not comfortable speaking to clinical limitations, as our background is as external evaluators of the program (as opposed to clinical experts). |
